# Identification of potential prognostic ceRNA module biomarkers in patients with pancreatic adenocarcinoma

**DOI:** 10.18632/oncotarget.21783

**Published:** 2017-10-10

**Authors:** Lili Zhao, Bingrong Liu

**Affiliations:** ^1^ Department of Gastroenterology, The Fourth Affiliated Hospital of Harbin Medical University, Harbin 150001, China; ^2^ Department of Gastroenterology, The Second Affiliated Hospital of Harbin Medical University, Harbin 150081, China

**Keywords:** hallmark gene, pancreatic adenocarcinoma, microRNAs, competitive endogenous RNA, long non-coding RNAs

## Abstract

Accumulating evidence suggested that long non-coding RNAs (lncRNAs) can function as competing endogenous RNAs (ceRNAs) to interact with other RNA transcripts and ceRNAs perturbation play important roles in cancer initiation and progression including pancreatic adenocarcinoma (PAAD). In this study, we constructed a PAAD-specific hallmark gene-related ceRNA network (HceNet) using paired genome-wide expression profiles of mRNA, lncRNA and miRNA and regulatory relationships between them. Based on “ceRNA hypothesis”, we analyzed the characteristics of HceNet and identified a ceRNA module comprising of 29 genes (12 lncRNAs, two miRNAs and 15 mRNAs) as potential prognostic biomarkers related to overall survival of patients with PAAD. The prognostic value of ceRNA module biomarkers was further validated in the train (Hazard Ratio (HR) =1.661, 95% CI: 1.275–2.165, p<1.00e-4), test (HR=1.546, 95% CI: 1.238-1.930, p<1.00e-4), and entire (HR=1.559, 95% CI: 1.321-1.839, p<1.00e-4) datasets. Our study provides candidate prognostic biomarkers for PAAD and increases our understanding of ceRNA-related regulatory mechanism in PAAD pathogenesis.

## INTRODUCTION

Pancreatic adenocarcinoma (PAAD) was ranked as the fourth and seventh most common cancer in the United States and China [1–3]. To date, almost all PAAD patients develop metastases that are likely to cause death. Some risk factors, such as smoking, age and chronic alcohol consumption, have been found to be associated with the risk of PAAD [4]. Most of patients with PAAD always have a poor prognosis with 5-year survival rates of less than 5% due to its invasiveness and rapid progression [5, 6]. Therefore, identifying novel molecular biomarkers will facilitate the improvement of diagnosis and prognosis prediction for PAAC. With tremendous advances in molecular biology, the molecular-level understanding of the pathogenesis, diagnosis and prognosis of PAAD have largely improved during the past years. The development and progression of PAAD have been shown to be induced by the abnormal expression of some oncogenes and tumor-suppressor genes [7], and the deregulation of some signaling pathways [8]. Several mRNA-based molecular signatures for PAAD prognosis have been uncovered over the last few decades, such as 62-gene signature in Collisson’s study [9], 36-gene signature in Haider’s study [10] and so on.

Many studies have suggested miRNA-mediated ceRNA regulatory mechanisms play crucial roles in the initiation and development of tumors, in which long non-coding RNAs (lncRNAs) act as sponges of miRNAs by competing with mRNAs, especially cancer hallmark-related genes. Many transcriptome research demonstrated that altered expression of lncRNAs is significantly associated with various complex diseases [11–14], implying their potential roles as diagnostic and prognostic markers in human cancers [14–25]. In a case, lncRNAs can participate in a competition with endogenous RNA (ceRNA) to regulate gene expression [26, 27]. LncRNAs can decrease the repression function of miRNA target genes by competing with miRNA target genes for the same miRNA [28, 29]. LncRNA-associated ceRNA regulatory network has been widely reported in humans and some other species [30]. For example, the pseudogene *PTENP1* can regulate the expression of tumor suppressor *PTEN* by competing for the common miRNAs [31]. Additionally, the two hallmark genes, *MAML1* and *MEF2C*, can act as sponges of *miR-133* to modulate the expression of muscle-specific lncRNA *linc-MD1* [32]. However, the prognostic value of lncRNA-associated ceRNA regulatory network remains unknown.

In the present study, we performed hierarchical clustering and survival analyses in patients to identify significant lncRNA-associated ceRNA biomarkers associated with PAAD prognosis. Our study will not only provide novel lncRNAs as candidate biomarkers for prognosis prediction for PAAD patients, but also deepen our understanding of the development and progression of PAAD.

## RESULTS

### Construction and analysis of PAAD-specific ceRNA network

**Figure 1:**
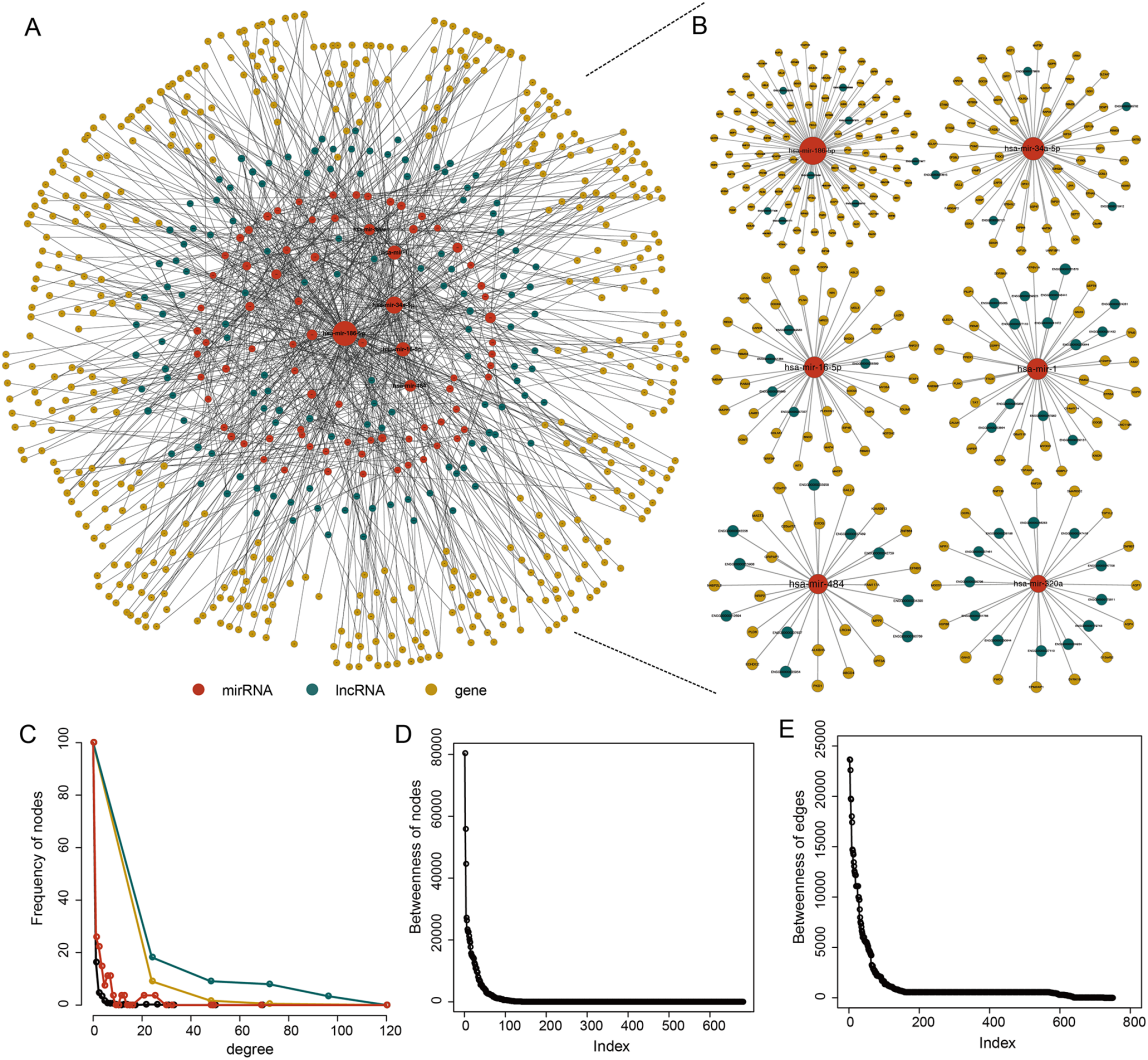
The PAAD-specific ceRNA network **(A)** The global PAAD-specific ceRNA network. **(B)** The top 6 miRNAs (miR-186-5p, miR-34a-5p, miR-16-5p, miR-1, miR-484, and miR-320a) ranked by node degree. **(C)** The degree distribution of the ceRNA network nodes. **(D)** The betweenness of the ceRNA network nodes. **(E)** The betweenness of the ceRNA network edges.

Based on “ceRNA hypothesis”, we first identified a total of 627 miRNA-mediated lncRNA-mRNA crosstalks in PAAD pathogenesis by integrating paired genome-wide expression profiles of mRNA, lncRNA and miRNA and regulatory relationships between them. Then these identified 627 miRNA-mediated lncRNA-mRNA crosstalks were integrated to construct a PAAD-specific ceRNA network, in which there were 1045 nodes (including 80 miRNAs, 128 lncRNAs and 473 mRNAs) and 1256 edges (Figure 1A and 1B) (Supplementary Table 1). The topological analysis indicated that the degree of nodes in PAAD-specific ceRNA network has a power law distribution (Figure 1C-1E). Further analysis suggested that some miRNA might play a key role in PAAD. For example, we listed the top 6 miRNAs (*miR-186-5p*, *miR-34a-5p*, *miR-16-5p*, *miR-1*, *miR-484*, and *miR-320a*) that had relatively high direct connections (degrees) in the network (Figure 1B), indicating that miRNAs played a hub role in connecting lncRNAs and mRNAs.

### Hallmark genes in the HceNet play critical roles in PAAD

**Figure 2:**
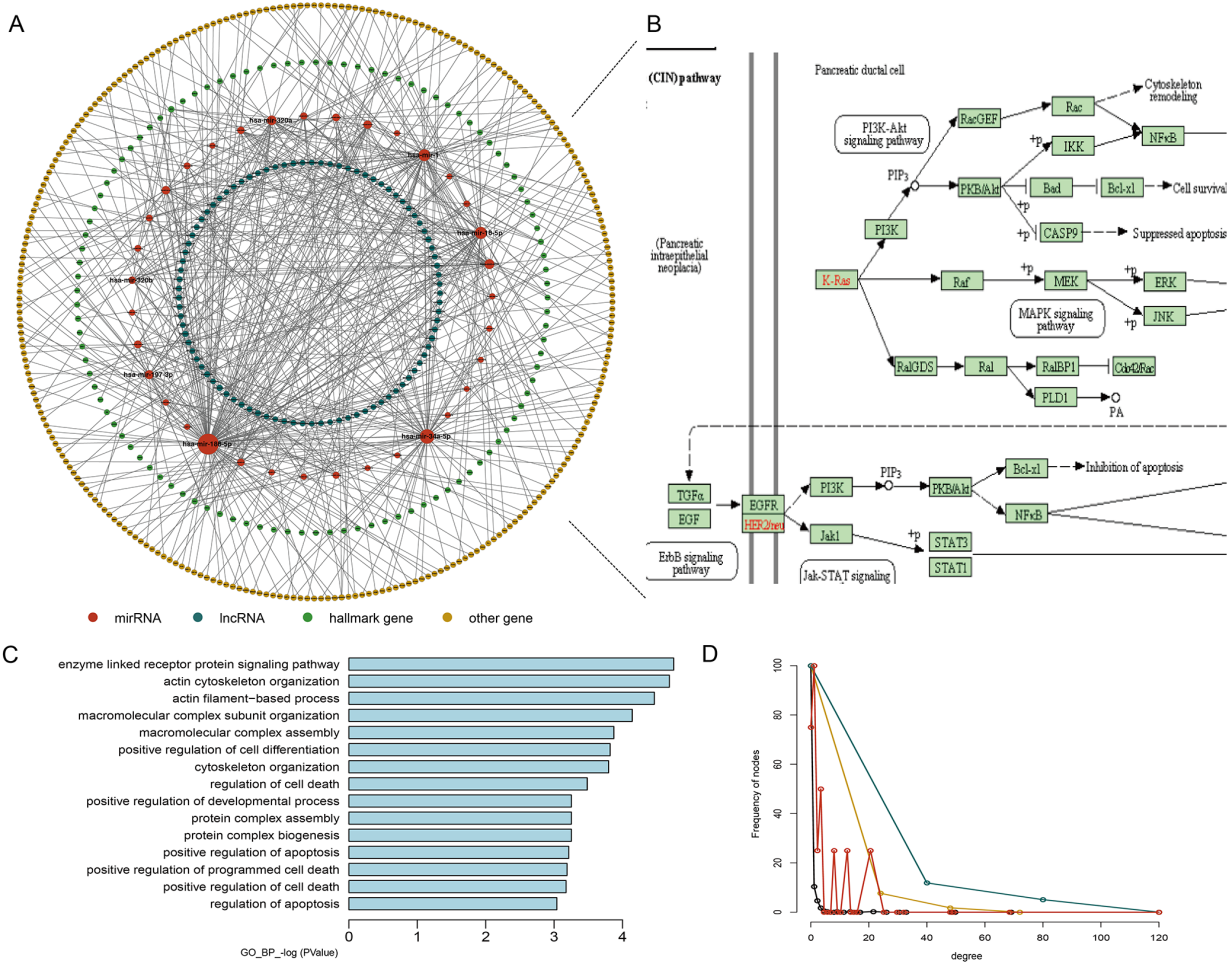
Properties of the hallmark gene-related ceRNA network (HceNet) **(A)** Global HceNet and the size of the sphere representing the degree of the node. **(B)** The functional PAAD subpathway. **(C)** The GO_FAT_BP terms for the HceNet genes. **(D)** The degree distribution of the different type nodes in HceNet.

According to a recent study, there are 7321 hallmark-related genes. Therefore, we constructed a hallmark gene-related ceRNA network (HceNet) (Figure 2A, Supplementary Table 2) from the ceRNA network. Next, we analyzed the function of the hallmark genes in the HceNet network and found that they were enriched in the KEGG pathway “PAAD” (Figure 2B). Moreover, we performed GO enrichment analysis using the genes in the HceNet (Figure 2C) and found some important GO terms associated with PAAD (i.e., “enzyme-linked receptor protein signalling pathway”, “regulation of T cell differentiation”, “positive regulation of cell differentiation”, “positive regulation of apoptosis”, and “positive regulation of programmed cell death”, etc.). We found many common genes in these GO terms that played key roles in the genesis and development of PAAD. For example, the common hallmark gene *DLC1* in several GO terms is an essential gene whose methylation in pancreatic ductal adenocarcinoma tissue samples is significantly associated with the stage, histological differentiation and lymph node metastasis [33]. Additionally, deficiency of the hallmark gene *NOTCH2* stops pancreatic intraepithelial neoplasia progression, prolongs survival, and results in a phenotypic switch towards anaplastic PAAD with the epithelial-mesenchymal transition [34]. *ETS-1* has been found to reduce cell migration and increase adhesion in pancreatic cancer cell lines by negatively correlating with E-cadherin expression [35]. Hence, our result will offer a direct mechanistic understanding of PAAD progression and greatly facilitate further experimental verification. Finally, topological analysis of the HceNet showed that the network displayed scale-free network characteristics regardless of the type of nodes (Figure 2D).

### Module analysis of PAAD-specific ceRNA network

**Figure 3:**
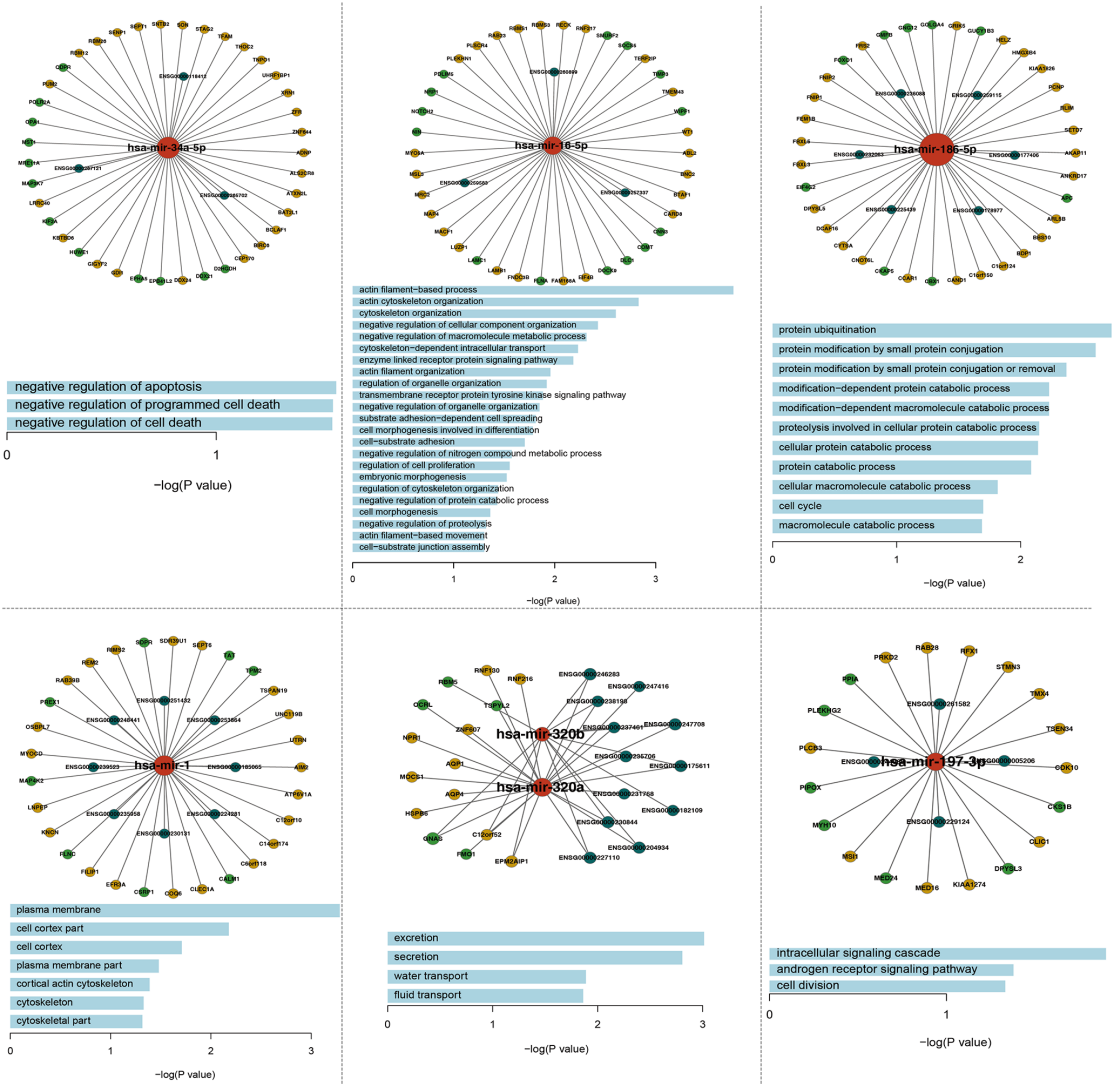
The six significant function modules and their enriched GO_FAT_BP terms

To investigate the relationships between miRNA-mediated ceRNA crosstalk and their functions, we performed a module analysis in the ceRNA network. We found six significant ceRNA modules (Figure 3, Supplementary Table 3) based on GraphWeb. Many genes and lncRNAs competitively combined with miRNAs in each module. For example, *DDX24*, *BAT2L1*, *ADNP*, *GDI1*, *CEP170*, *EPB41L2*, *BIRC6* and *D2HGDH* competitively combined with *miR-34a* with three lncRNA genes (ENSG00000265702, ENSG00000118412 and ENSG00000267121) in module 1. Furthermore, we conducted function enrichment analyses of the module genes based on GO terms using DAVID. With a cut-off P value of < 0.05, we identified some biological process terms that were significantly enriched with the module genes (Figure 3), including “negative regulation of apoptosis”, “cellular macromolecule catabolic process”, “intracellular signaling cascade”, “negative regulation of growth”, “negative regulation of programmed cell death”, and “cell cycle”. In previous studies, the GO term “negative regulation of programmed cell death” in the first and fifth modules was a common and essentially biological process for cell death and participated in the process of cancer development. In this GO term, several genes (*MAP3K7*, *SON*, *ADNP*, *BIRC6*, *RNF130*, *RBM5* and *RNF216*) may take part in PAAD. Previous study has demonstrated the critical roles of SON in the proliferation, tumorigenicity and survival of PAAD cells, implying that *SON* may be a candidate therapeutic target for PAAD [36]. Moreover, Peng J and others found that dysregulated expression of *RBM5* was significantly associated with poor clinicopathological features of PAAD [37].

### Identification of potential ceRNA module biomarkers in PAAD

**Figure 4:**
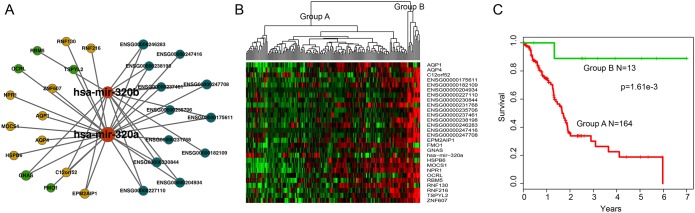
Overview of 12-lncRNA, two-miRNA and 15-mRNA module and its prognostic ability for assessing the clinical outcome of PAAD **(A)** Overview of the 12-lncRNA, two-miRNA and 15-mRNA module. **(B)** Hierarchical clustering heatmap and dendrogram of patients based on the expression patterns of module molecules in PAAD. **(C)** KM survival curves of two subgroup patients resulted from the unsupervised hierarchical clustering in PAAD. P value was calculated using the log-rank test.

Based on GraphWeb module mining strategy, six modules were identified in HceNet. In order to assess the predictive power of each module for patients’ survival, we performed an unsupervised hierarchical clustering analysis. The result showed that 177 patients were clustered into two major groups (13 patients vs. 164 patients) based on the expression pattern of genes (12-lncRNAs, two-miRNAs and 15-mRNAs) in the fifth module (Figure 4A and 4B). Moreover, there is a significant difference in overall survival (OS) between these two patient subgroups (log-rank test p=1.61e-03; Figure 4C), implying the roles of this module as candidate molecular biomarker for survival prediction in PAAD. For instance, previous studies have shown that *GNAS* mutations can provide a help in the risk stratification and surveillance of patients with pancreatic cancer [38, 39], which may inform the diagnosis and management of PAAD.

### Prognostic performance evaluation of ceRNA module biomarkers in PAAD

**Figure 5:**
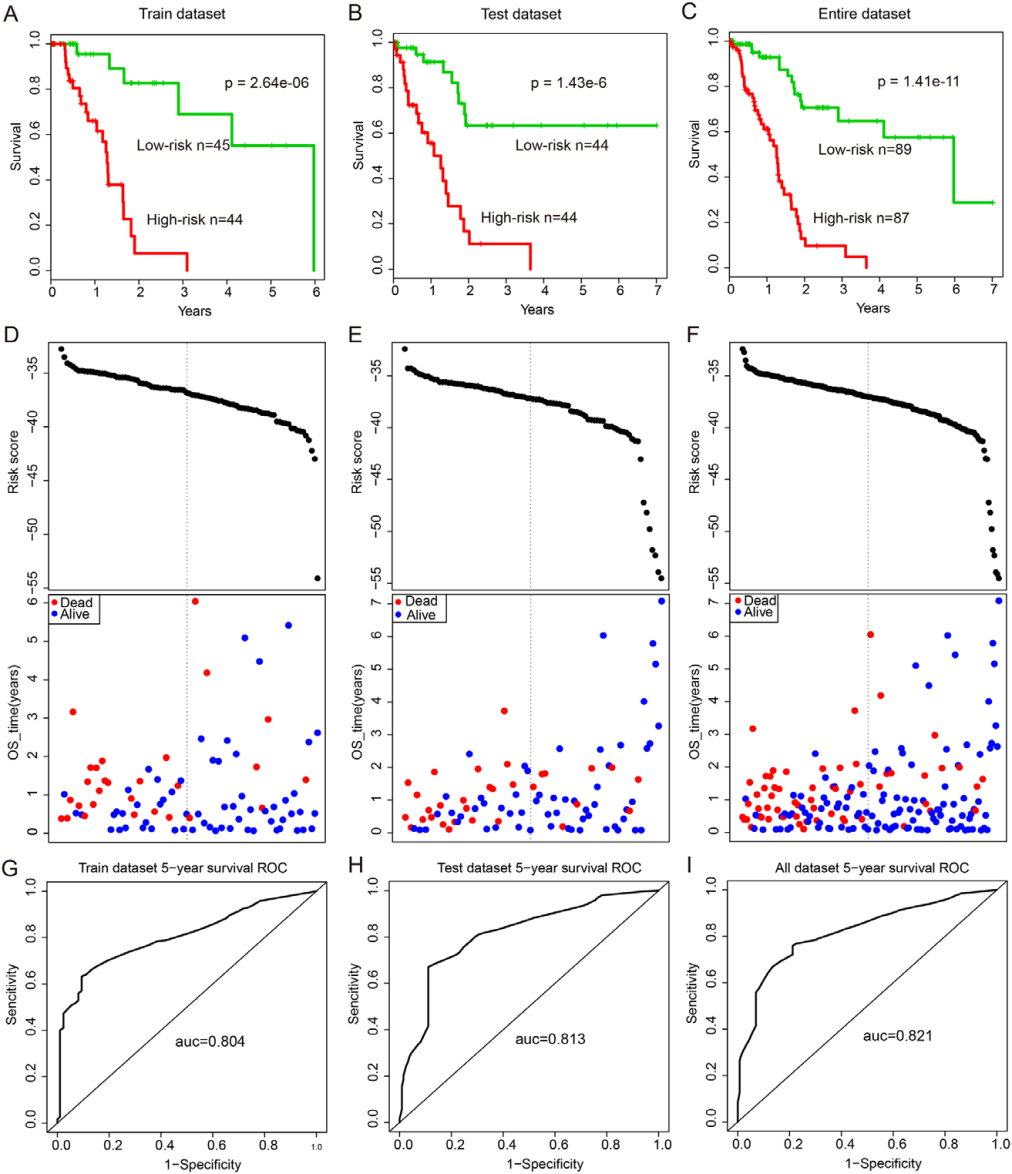
Survival curves and risk score analysis of the 12-lncRNA, two-miRNA and 15-mRNA module in train, test, and entire datasets, respectively **(A-C)** KM survival curves for disease free survival of patients in the train, test and entire datasets respectively with high and low-risk scores. P value was calculated using the log-rank test. **(D-F)** Risk score analysis of the module in train, test and entire datasets patients respectively. **(G-I)** Receiver operating characteristic (ROC) curve analysis and area under the curve (AUC) value of the ROC curve indicating the sensitivity and specificity of the module for survival prediction.

To further validate the predictive power of this ceRNA module biomarker, expression data of 29 genes (12-lncRNAs, two-miRNAs and 15-mRNAs) in this ceRNA module were fitted in a multivariate Cox regression analysis. Then a risk score predictive model was developed as described in Methods (Supplementary Table 4). All 177 patients in this study were randomly divided into two patient datasets: training dataset (n=89) and test dataset (n=88). Firstly, the risk score model was applied to the training dataset in which 89 patients were classified into high-risk (n=44) and low-risk groups (n=45) using the median risk score (-37.16) as the cut-off. The two groups of patients revealed significantly different survival times (log-rank p=2.64e-3, Figure 5A). Moreover, the risk score model revealed similar prognostic power in the test dataset (log-rank p=1.43e-6, Figure 5B) and the entire dataset (log-rank p=1.41e-11, Figure 5C). Results of univariate Cox analysis also indicated the significant association between the risk score and patients’ survival in the training (Hazard Ratio (HR)=1.661, 95% CI: 1.275–2.165, p<1.00e-4), test (HR=1.546, 95% CI: 1.238-1.930, p<1.00e-4), and entire (HR=1.559, 95% CI: 1.321-1.839, p<1.00e-4) datasets (Table 1). The distribution of risk scores and patients’ survival status in these three datasets are presented in Figure 5D-5F. Further time-dependent receiver operating characteristic (ROC) curve analysis suggested that the risk score model achieved the area under the curve (AUC) values of 0.804, 0.813 and 0.821 in the training, test and entire datasets, respectively (Figure 5G-5I).

**Table 1:** Univariate and multivariate Cox regression analysis of the ceRNA module biomarkers in PAAD patients

Variables	Univariable model			Multivariable model		
	HR	95% CI of HR	P-value	HR	95% CI of HR	P-value
Train dataset						
Risk score	1.661	1.275-2.165	<0.0001	1.587	1.168-2.155	0.003
Gender	1.801	0.784-4.136	0.166	1.870	0.737-4.746	0.188
Age	1.012	0.974-1.051	0.539	1.006	0.971-1.042	0.741
Grade						
G2	3.496	0.778-15.713	0.103	0.921	0.169-5.032	0.924
G3	4.848	1.021-23.022	0.047	1.670	0.306-9.105	0.553
Stage						
IIA	1.821	0.211-15.746	0.586	2.226	0.231-21.496	0.489
IIB	2.789	0.371-20.982	0.319	3.510	0.419-29.400	0.247
Test dataset						
Risk score	1.546	1.238-1.930	<0.0001	1.876	1.379-2.552	<0.0001
Gender	0.858	0.423-1.739	0.671	0.971	0.416-2.263	0.945
Age	1.043	1.008-1.079	0.017	1.025	0.988-1.064	0.191
Grade						
G2	2.556	0.732-8.923	0.141	0.464	0.114-1.888	0.284
G3	4.681	1.301-16.838	0.018	1.855	0.456-7.542	0.388
Stage						
IIA	1.723	0.285-10.437	0.554	0.162	0.024-1.105	0.063
IIB	4.380	1.281-14.972	0.019	0.272	0.063-1.180	0.082
Entire dataset						
Risk score	1.559	1.321-1.839	<0.0001	1.562	1.307-1.868	<0.0001
Gender	1.262	0.747-2.132	0.385	1.291	0.729-2.286	0.382
Age	1.032	1.006-1.058	0.015	1.017	0.992-1.043	0.179
Grade						
G2	3.301	1.268-8.588	0.014	0.847	0.301-2.386	0.753
G3	5.158	1.913-13.913	0.001	1.464	0.520-4.124	0.471
Stage						
IIA	2.524	0.754-8.448	0.133	0.763	0.216-2.702	0.675
IIB	4.279	1.511-12.116	0.006	1.337	0.468-3.824	0.587

**Figure 6:**
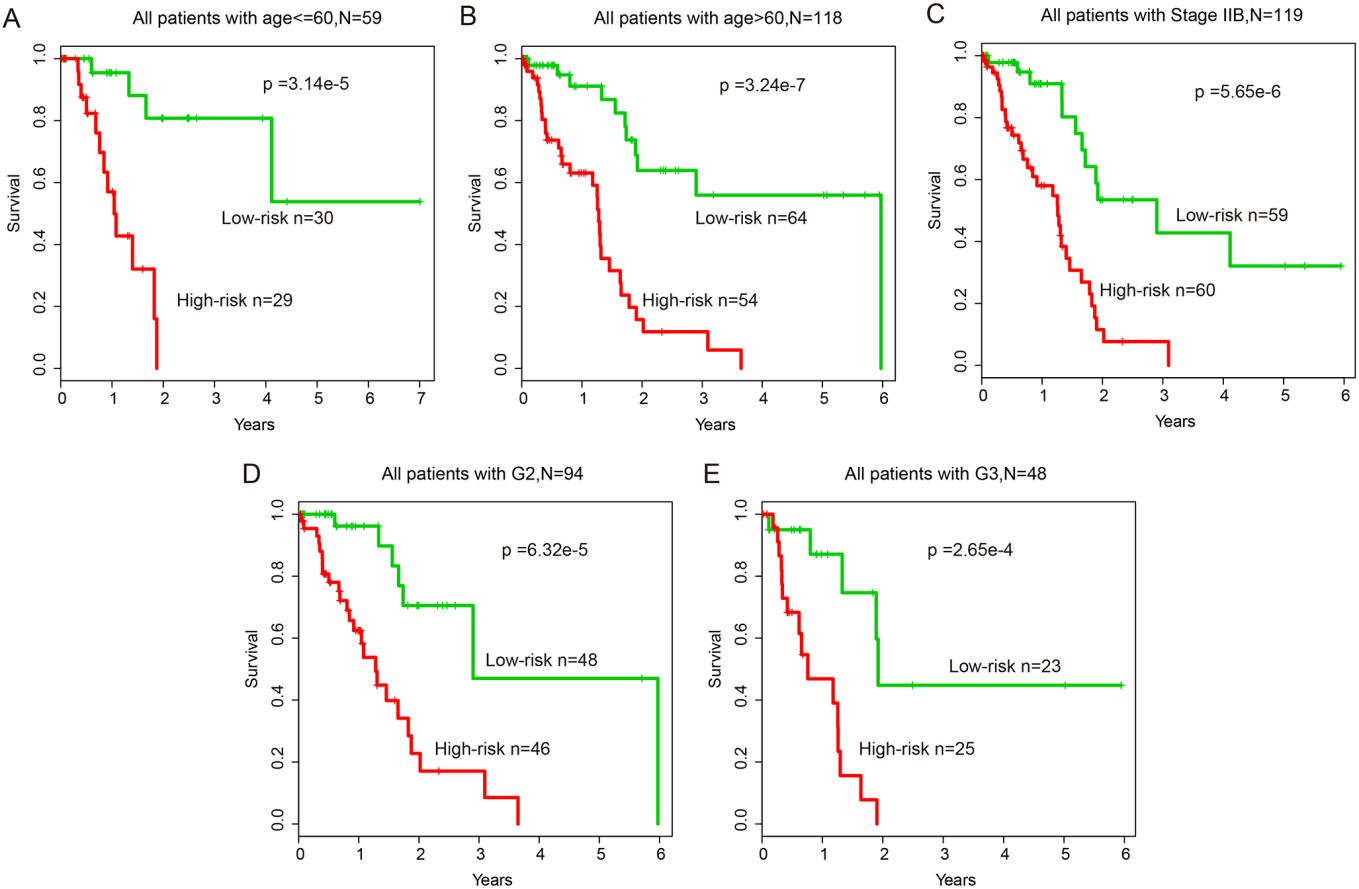
Stratification analysis of all patients based on age, grade and stage **(A-B)** KM survival curves of patients stratified by age based on the 12-lncRNA, two-miRNA and 15-mRNA module (age <= 60, N = 59; age > 60, N = 118). **(C)** KM survival curves of patients stratified by stage IIB based on the 12-lncRNA, two-miRNA and 15-mRNA module (N = 119). **(D-E)** KM survival curves of patients stratified by grade based on the 12-lncRNA, two-miRNA and 15-mRNA module (G2, N = 94; G3, N = 48).

In addition, multivariate analysis and stratification analysis was carried out in order to assess the independence of the risk score model with respect to other clinical features. Results of multivariate analysis showed that the risk score model still has a significant association with OS after adjusting for other clinical features in training (HR=1.587, 95% CI: 1.168-2.155, p=3.00e-3), test (HR=1.876, 95% CI: 1.379-2.552, p<1.00e-4) and entire (HR=1.562, 95% CI: 1.307-1.868, p<1.00e-4) datasets (Table 1). Stratification analysis indicated that the risk score model is independent of age, grade and stage, as it performed equally well in different age groups (log-rank test p=3.14e-5 for patients <=60 years and p=3.24e-7 for patients >60 years), in patients with stage IIB (log-rank test p=5.65e-6) and in patients with grade G2 or G3 (log-rank test p=6.32e-5 for patients with G2 and p=2.65e-4 for patients with G3) (Figure 6A-6E).

## DISCUSSION

Progresses in omics have revealed a complex genomic landscape of PAAD, including point mutations, gene expression aberration and so on [40]. PAAD is an aggressive malignancy with a high 5-year mortality rate [41]. Recently, a variety of molecular biomarkers in PAAD has been identified [42, 43]. In our study, we explored the mechanism underlying PAAD and sought to identify new diagnostic markers for this disease.

Recently, many human ceRNAs in RNA regulatory networks have been identified as competitors in binding the common miRNA [44–46]. However, prognostic value of ceRNA module has not been systematically examined in PAAD. Despite the fact that the biogenesis of PAAD is extremely complex, the complexity of tumor growth and metastasis dissemination can be largely represented by several cancer hallmarks [47], In this study, a transcriptome-wide miRNA-mediated lncRNA-mRNA ceRNA network was constructed to provide a comprehensive resource for investigating the ceRNA regulation in human PAAD by cancer hallmark genes. Additionally, hallmark genes in the ceRNA network have been found to exert crucial ceRNA roles by acting as miRNA sponges [48]. Some previous studies found that miRNA-mediated ceRNA regulations are involved in broad cancer-related hallmarks and suggest that hallmark-related genes exhibit important regulated roles in the ceRNA interaction pairs [49, 50].

The TCGA project has provided comprehensive transcriptome data identified in tumor and matched normal samples [51], thereby enabling the identification of miRNA-mediated lncRNA-mRNA crosstalks associated with PAAD. In this study, we performed a systematic analysis of PAAD-specific ceRNA network and developed a network-based strategy to identify the ceRNA interaction network and modules. Many hallmark genes played vital roles in the prognostic module; thus, this module might serve as a survival-associated biomarker for PAAD. Our results also revealed the underlying mechanism of the LMceCTs that involved in the dysregulation of hallmark cancer genes.

In summary, the ceRNA network offered a novel insight to explore the disturbed competitive interactions among RNAs in PAAD. These findings will provide candidate prognostic biomarkers for PAAD and improve our understanding of ceRNA-related regulatory mechanism in PAAD pathogenesis.

## MATERIALS AND METHODS

### Data collection

The GENCODE (V19) annotation file in GTF format was used to obtain lncRNAs. Original Ensembl transcript names were used as identifiers for the different lncRNAs. The dataset contained a total of 12,905 lncRNA genes.

The pancreatic adenocarcinoma (PAAD) RNASeqV2 level 3 and clinical datasets were derived from The Cancer Genome Atlas (TCGA, http://cancergenome.nih.gov/). The mRNA expression profile data were obtained from the above data, and lncRNA expression profile data of PAAD patients were obtained by repurposing the exome sequencing data from RNASeqV2 level 3. Briefly, the exome sequencing data sets were re-annotated to the human genome. Then those exons that uniquely mapped to lncRNA sequences were kept to represent lncRNAs. The expression levels of lncRNAs were obtained by background correction and quantile normalization [52]. Finally, expression profiles of 20,072 mRNAs, and 2,710 lncRNAs in 177 PAAD patients with clinical information were enrolled in our study.

Human miRNAs and their experimentally validated targets mRNAs and lncRNAs were downloaded from miRTarBase (version 6.1) [53], starBase v2.0 [54] and DIANA-LncBase [55]. Finally, a total of 322,135 non-redundant miRNA-mRNAs interactions and 106,496 miRNA-lncRNA interactions were obtained for further analysis

The hallmark-associated gene set was obtained from MsigDB V5.1, which is a collection of annotated gene sets for use with the GSEA software [56]. Finally, a total of 7321 hallmark-associated genes were obtained for subsequent network construction and topological feature analyses.

### Construction of the PAAD-specific ceRNA network

Using the experimentally supported miRNA–mRNA and miRNA-lncRNA regulatory data, we primarily followed the two principles listed below to construct PAAD-specific ceRNA network. A central principle of our hypothesis was that trans-regulatory ceRNA crosstalk increased with the high miRNA regulatory similarity and co-expression between mRNAs and lncRNAs. First, a hypergeometric test was performed for each possible lncRNA and mRNA pair separately. For each given pair of A and B, we identified the common miRNA that regulated them (A ∩ B). Then, the probability P for A and B was calculated according to

$$\mathrm{P=1}-F\left(x|\mathrm{N,K,M}\right)=1-\sum_{t=0}^{x}\frac{\left(\begin{array}{c} K\\ t \end{array}\right)\left(\begin{array}{c} N-K\\ M-t \end{array}\right)}{\left(\begin{array}{c} N\\ M \end{array}\right)}$$

where N was the number of all miRNAs, K and M were the total numbers of miRNAs regulated by A and B, and x was the common miRNA numbers between these pairs. Only pairs regulated by at least one common miRNA were analyzed in our study. Candidate lncRNA-mRNA pairs with P-values less than 0.05 were used for the subsequent analysis. Next, to explore the active ceRNA pairs in PAAD, we computed the correlation coefficient (R) for each candidate ceRNA pair identified above. All Pearson candidate pairs with R>0.5 and p-adjusted<0.05 were identified as ceRNA-ceRNA interactions. LncRNA–mRNA pairs interacting with the same miRNA were defined as ceRNA crosstalk.

### Topological measurements of the ceRNA network

For each miRNA in a network, the degree is defined as the number of edges to which it is connected. Hub miRNA with higher degrees in biological networks are most likely to essential. Thus, in this work we selected the top six miRNAs with the highest degrees in the ceRNA network as the hubs. We also analyzed other topological attributes of the network, such as the degree of each node type and the betweenness of the nodes and edges.

### Construction of the hallmark gene-related ceRNA network

The hallmark gene-related ceRNA network (HceNet) was built by mapping the hallmark genes to the PAAD-specific ceRNA network. Then, edges linked by the hallmark genes were extracted. Similarly, we analyzed the degree distribution of the HceNet. The networks were visualized using Cytoscape 3.0.2 [57] and the topology analysis was performed by the ‘igraph’ package in the R language.

### Functional enrichment analysis

Functional enrichment analysis at the GO and KEGG levels was performed using DAVID Bioinformatics Resources (http://david.abcc.ncifcrf.gov/, version 6.7) [58]. The DAVID enrichment analysis was limited to KEGG pathways and GO-FAT biological process (BP) terms with the whole human genome as background. Functional categories with a p-value of < 0.05 were considered statistically significant and were visualized using R language.

### Identification of ceRNA modules

By using the HceNet constructed in the above step, we identified the network modules with GraphWeb [59], which is a web server used to identify network-based biomarkers that most represent the property of the network. Then, the web server was applied to the interacting pairs in the HceNet and the most significant modules were retained in our analysis. Additionally, in order to mine the significantly survival-associated module, we performed gene expression hierarchical clustering analysis for the identified modules and explored whether they could distinguish the PAAD patients.

### Survival analysis

To verify whether the module we identified in the above step was associated with PAAD patient survival, we extracted the expression of the lncRNAs and mRNAs in the module. Then, Cox proportional hazard analysis was used to obtain the regression coefficient of each gene associated with patients’ survival. The classifier was built as the linear combination of the gene expression values of the selected genes using the standardized Cox regression coefficient as the weight. A risk score formula for each patient was established by including the expression values of each selected gene weighted by their estimated regression coefficients in the multivariate Cox regression analysis. Finally, patients were divided into high-risk and low-risk groups [60] using the median of the risk score as the threshold. Kaplan-Meier survival plots and log-rank tests were used to assess the differences in overall survival (OS) times between the high-risk and low-risk patients. In addition, we evaluated the sensitivity and specificity of the module for survival prediction using receiver operating characteristic (ROC) curve analysis and the area under the ROC curve (AUC).

## SUPPLEMENTARY MATERIALS TABLES










