# Supplementary material for: Identification of potential prognostic ceRNA module biomarkers in patients with pancreatic adenocarcinoma
Source: Oncotarget. 2017 Oct 10;8(55):94493–504. doi: 10.18632/oncotarget.21783 (PMC5706890; doi:10.18632/oncotarget.21783)
Supplement: Supplementary file 1 [file oncotarget-08-94493-s001.pdf]

Identification of potential prognostic ceRNA module biomarkers in patients with pancreatic adenocarcinoma

SUPPLEMENTARY MATERIALS

Supplementary Table 1: The Catalog of LMceCTs

See Supplementary File 1

Supplementary Table 2: Hallmark gene-related ceRNA network

See Supplementary File 2

Supplementary Table 3: The six significant function modules

See Supplementary File 3

Supplementary Table 4: The Cox regression coefficients of the prognostic module moleculars

See Supplementary File 4
